# Propensity score-matched comparison of total arterial off- and on-pump coronary artery bypass with complete revascularization

**DOI:** 10.1007/s00380-023-02317-x

**Published:** 2023-09-25

**Authors:** Shizuya Shintomi, Satoshi Saito, Azumi Hamasaki, Yuki Ichihara, Kozo Morita, Masahiro Ikeda, Satoru Domoto, Akihisa Furuta, Hiroshi Niinami

**Affiliations:** Department of Cardiovascular Surgery, Tokyo Women’s Medical University Hospital, 8-1, Kawada-cho, Shinjuku, Tokyo Japan

**Keywords:** Total arterial coronary artery bypass grafting, Complete revascularization, Propensity score matching, Long-term outcomes, Risk factors

## Abstract

Little is known regarding the long-term (> 10 years) outcomes and risk factors of total arterial coronary artery bypass grafting (CABG). This study evaluated the long-term outcomes and risk factors for all-cause mortality and major adverse cardiac and cerebrovascular events (MACCEs) following total arterial on-pump CABG (ONCAB) or off-pump CABG (OPCAB) with complete revascularization. This retrospective cohort analysis enrolled patients with stable angina who underwent total arterial CABG with complete revascularization in our institute between July 2000 and June 2019. The endpoints were all-cause mortality and MACCE incidence, including a comparison between OPCAB and ONCAB. Long-term (10-year) outcomes were analyzed using propensity score-matched pairs, and risk factors were evaluated using univariate and multivariate analyses. Overall, 401 patients who underwent primary total arterial CABG were classified into the OPCAB (*n* = 269) and ONCAB (*n* = 132) groups. Using propensity score matching (PSM), 88 patients who underwent OPCAB were matched with 88 patients who underwent ONCAB. The mean follow-up period was 7.9 ± 6.3 years. No significant difference in all-cause mortality (hazard ratio, 1.04; 95% confidence interval, 0.53–2.04; *p* = 0.9138) and MACCE incidence (hazard ratio, 1.06; 95% confidence interval, 0.68–1.65; *p* = 0.7901) was observed between the two groups. Renal failure requiring dialysis was a significant risk factor for mortality (*p* < 0.0001) and MACCEs (*p* = 0.0003). Long-term outcomes of total arterial OPCAB and ONCAB with complete revascularization showed similar findings using PSM. Renal failure requiring dialysis was a significant risk factor for mortality and morbidity.Journal standard instruction requires an unstructured abstract; hence the headings provided in abstract were deleted. Kindly check and confirm.Thank you for your kindness.

**Clinical registration number** 5598, Tokyo Women’s Medical University Hospital.

## Introduction

The SYNTAX trial showed that coronary artery bypass grafting (CABG) is more effective than percutaneous coronary intervention (PCI) in complex coronary artery regions [1–3].

It is also apparent that failure rates are predominantly higher with saphenous vein grafts (SVG) than with arterial grafts in terms of perioperative outcomes [4, 5]. Furthermore, CABG using arterial grafts could improve mid-term survival (up to 10 years) [6–8].

However, there is limited information regarding the long-term (> 10 years) outcomes and risk factors for total arterial CABG. Furthermore, comparisons of outcomes between total arterial off-pump CABG (OPCAB) and on-pump CABG (ONCAB) with complete revascularization have not been reported often.

Thus, this study aimed to assess the clinical benefits of total arterial OPCAB compared with those of ONCAB with complete revascularization using propensity score matching (PSM) and to reveal the risk factors for total arterial CABG. Additionally, the study examined the 30-day complications, details of postoperative graft patency, and risk factors for mortality and MACCEs.

## Materials and methods

### Study design

This study was a retrospective cohort analysis of patients with stable angina who underwent total arterial CABG with complete revascularization in our institute between July 2000 and June 2019. The inclusion and exclusion criteria for selecting the study participants are summarized in Table 1. Younger patients underwent total arterial CABG for long-term patency.

**Table 1:** Inclusion and exclusion criteria

Inclusion criteria
1. Significant stenosis by preoperative angiography
2. Graftable target vessels
3. Available grafts
Exclusion criteria
1. Vein graft usage
2. Redo
3. Concomitant procedure
4. Acute myocardial infarction
5. MIDCAB
6. Single revascularization

*MIDCAB* minimally invasive direct coronary artery bypass grafting

Please check and confirm the layout of all tables.Thank you. I have checked and confirmed that everything is in order

The preoperative risk score was calculated based on patients’ characteristics. Postoperative coronary angiography was routinely performed on patients depending on their renal function. All patients were administered selective graft injections.

The Institutional Review Board of Tokyo Women’s Medical University approved this study (Approval No. 5598). The review board waived the need for informed consent because of the retrospective nature of this study. This study was performed in conformance with the Declaration of Helsinki.

### Operation

ONCAB was performed previously at the facility. Recently, depending on the surgeon’s preference, OPCAB is now the preferred option. Both procedures were performed by several surgeons. Prophylactic intra-aortic balloon pumping (IABP) was performed for patients with severe left main trunk disease or a low left ventricular ejection fraction (LVEF).

All arterial grafts were harvested in a skeletonized manner using an ultrasonic scalpel (Harmonic Scalpel; Ethicon Endosurgery, Cincinnati, OH). The left internal thoracic artery (LITA), right internal thoracic artery (RITA), and gastroepiploic artery (GEA) were used as in-situ grafts. The radial artery (RA) was anastomosed to the ascending aorta as central anastomosis and in-situ grafts were divided after heparinization. The bilateral internal thoracic artery and in-situ gastroepiploic artery (GEA) were the preferred in-situ grafts with OPCAB to achieve total arterial revascularization.

### Surgical technique for OPCAB

A stabilizer (Octopus, Medtronic, Minneapolis, MN, USA) was used for the heart. Two deep pericardial sutures were placed in the posterior pericardium between the inferior vena cava and the left lower pulmonary vein, exposing the lateral or inferior walls. A bloodless field was acquired using a proximal snare and a carbon dioxide blower. Each anastomosis was performed using an 8-0 polypropylene running suture with the parachute technique. The graft patency was assessed using transit-time flow measurement before and after protamine reversal.

### Surgical technique for ONCAB

After preparing the conduits, standard cannulation for cardiopulmonary bypass was performed using ascending aortic cannulation and dual-stage cannulation of the right atrium with an antegrade cardioplegia cannula. The aorta was clamped, and cold blood cardioplegia was injected every 20 min. After completing the anastomosis, the patient was weaned off cardiopulmonary bypass. Similarly, graft patency was assessed using transit-time flow measurement before and after protamine reversal.

### Postoperative coronary angiography

Postoperative coronary angiography was performed around 1–2 weeks postoperatively before discharge. The exclusion of postoperative coronary angiography was determined by considering patients’ renal function and general condition.

### Definition of complete revascularization

Complete revascularization was defined as the treatment of any lesion with > 75% area stenosis in vessels measuring ≥ 1.0 mm, as estimated on the diagnostic angiography.

### Study outcomes

The primary endpoint was all-cause mortality, including the comparison between OPCAB and ONCAB. In contrast, the secondary endpoint was the incidence of MACCEs.

### Statistical analysis

Continuous variables are reported as mean ± standard deviation and number (%) and were analyzed using an unpaired *t*-test or Mann–Whitney *U* test. Categorical variables were analyzed using Pearson’s Chi-squared test. Survival curves were drawn on an actuarial basis using the Kaplan–Meier technique, and comparisons were made using Cox proportional hazard ratios and the log-rank test. Univariate and multivariate Cox proportional hazard regression models were constructed to identify independent predictors of mortality and MACCEs.

Considering the differences between the baseline characteristics of the two groups, PSM was used to identify a cohort of patients with similar baseline characteristics, matched for age, male sex, body mass index, old myocardial infarction, previous PCI, previous stroke, peripheral arterial disease, renal failure requiring dialysis, hypertension, diabetes mellitus, diabetes mellitus with insulin, LVEF, LVEF < 35%, IABP, left main coronary disease, triple vessels coronary disease, JapanSCORE, euroSCORE II, Society of Thoracic Surgeons score, and SYNTAX score.

The propensity score was estimated with a non-parsimonious multivariable logistic regression model using all the baseline characteristics outlined in Table 1 as covariates. Matching was performed using a 1:1 matching protocol without replacement (greedy-matching algorithm), with a caliper width equal to 0.2 of the standard deviation of the logit of the propensity score.

A two-sided *p-*value of < 0.05 was considered statistically significant for all tests. All statistical analyses were performed using JMP Pro version 15 (SAS Institute Inc, Cary, NC, USA) software.

## Results

### Patient population

**Fig. 1:**
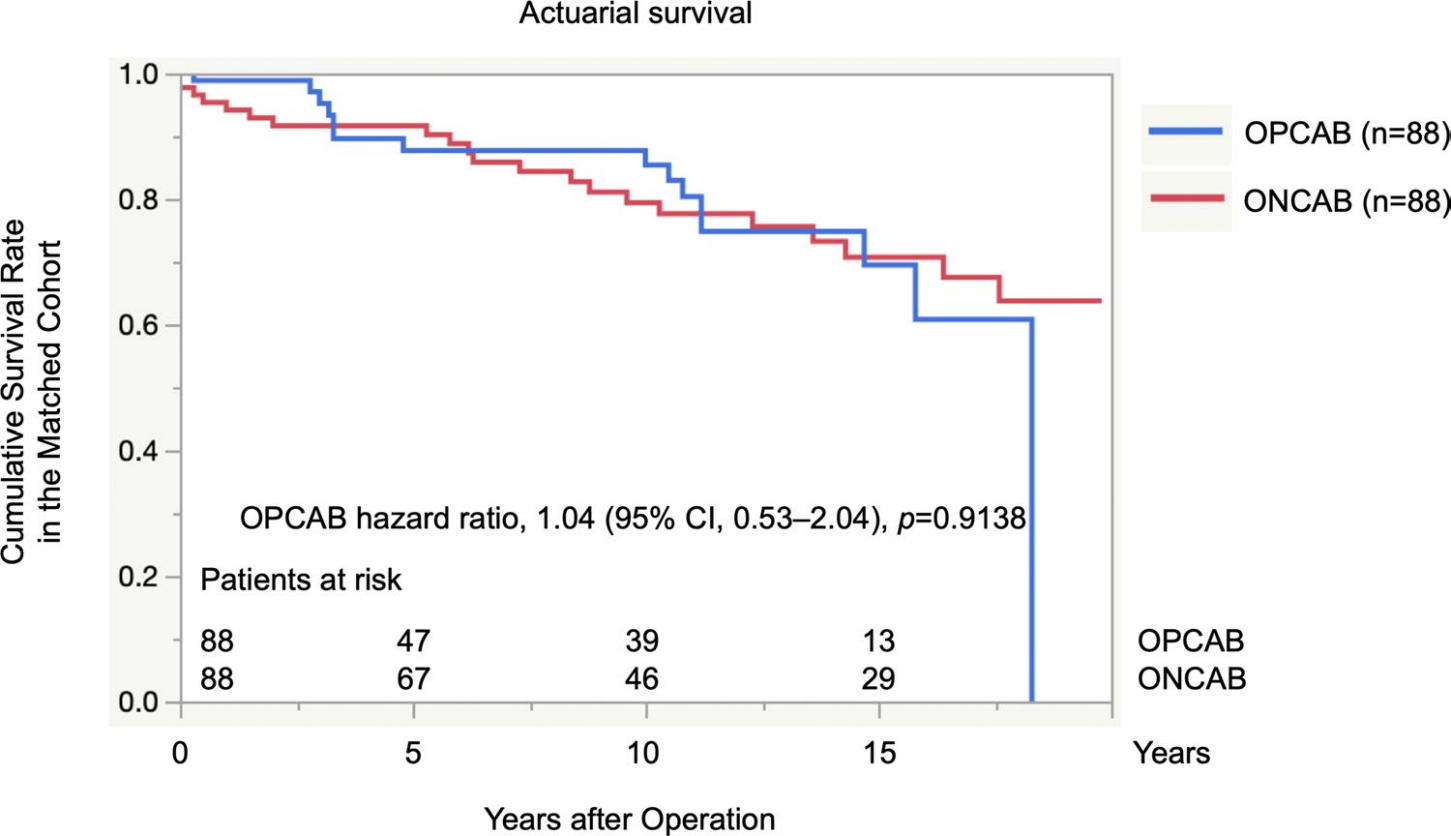
Cumulative survival rates in the matched cohort. The panel shows the survival curves of the matched cohort. The hazard ratios of the off-pump group compared to those of the on-pump group are shown. *OPCAB* off-pump coronary artery bypass grafting, *ONCAB* on-pump coronary artery bypass grafting

In total, 1498 patients underwent primary CABG in our institute. This study enrolled 401 of them who underwent primary total arterial CABG. All patients underwent complete revascularization. OPCAB and ONCAB were performed on 269 and 132 patients, respectively (Fig. 1). Although patients who underwent OPCAB and ONCAB were randomly, not sequentially, grouped, the number of OPCAB procedures in the facility has increased recently. Patients who underwent combined operations were excluded.

### Preoperative characteristics

The preoperative characteristics of the patients are shown in Table 2. Before PSM, significant differences in the incidence of old myocardial infarction (OMI), hypertension (HT), and left ventricular ejection fraction (LVEF) ≤ 35% were observed between the two groups. Using PSM, 88 patients who underwent OPCAB were matched with 88 patients who underwent ONCAB.

**Table 2:** Operative characteristics

	All (*n* = 401)	Unmatched			Matched		
		OPCAB (*n* = 269)	ONCAB (*n* = 132)	*p*-value	OPCAB (*n* = 88)	ONCAB (*n* = 88)	*p*-value
Age	63.5 ± 9.9	63.6 ± 9.8	63.3 ± 10.0	0.7722	64.3 ± 9.2	63.1 ± 10.4	0.6741
Male	340 (85)	226 (84)	114 (86)	0.5383	75 (85)	74 (84)	0.8343
BMI	24.3 ± 3.2	24.4 ± 3.3	23.9 ± 2.9	0.2728	23.8 ± 3.2	23.8 ± 3.0	0.7027
Clinical history							
OMI	174 (43)	96 (36)	78 (59)	< 0.0001	45 (51)	48 (55)	0.6505
PCI	75 (19)	53 (20)	22 (16)	0.4638	15 (17)	15 (17)	1.0000
Stroke	40 (10)	23 (9)	17 (13)	0.1741	13 (15)	11 (13)	0.6604
PAD	25 (6)	15 (6)	10 (8)	0.4364	5 (6)	6 (7)	0.7555
HD	53 (13)	40 (15)	13 (10)	0.1630	9 (10)	10 (11)	0.8081
HT	182 (45)	111 (41)	71 (54)	0.0179	52 (59)	49 (56)	0.6475
DM	208 (52)	140 (52)	68 (51)	0.9206	45 (51)	49 (56)	0.5456
Insulin	27 (7)	18 (7)	9 (7)	0.9620	6 (7)	5 (6)	0.7555
DL	166 (41)	104 (39)	62 (47)	0.1125	40 (45)	40 (45)	1.0000
LVEF	50.5 ± 11.7	51.5 ± 10.5	48.2 ± 13.9	0.0529	50.8 ± 11.8	49.8 ± 13.4	0.5528
≤ 35%	56 (14)	30 (11)	26 (20)	0.0204	13 (15)	15 (17)	0.6802
IABP	88 (22)	59 (22)	29 (22)	0.9934	16 (18)	19 (22)	0.5710
Diseased vessels							
LMT	81 (20)	59 (22)	22 (17)	0.2171	16 (18)	15 (17)	0.8431
TVD	218 (54)	144 (53)	74 (56)	0.6328	44 (50)	46 (52)	0.7630
Preoperative risk score							
Japan-SCORE	1.3 ± 1.4	1.4 ± 1.5	1.1 ± 1.0	0.1229	1.1 ± 0.8	1.2 ± 1.1	0.6410
Euro-SCORE II	1.4 ± 1.0	1.4 ± 0.9	1.5 ± 1.3	0.2612	1.4 ± 1.0	1.5 ± 1.2	0.7269
STS score	1.0 ± 0.7	1.0 ± 0.7	1.0 ± 0.7	0.8279	1.0 ± 0.7	1.0 ± 0.7	0.7672
SYNTAX score	23.3 ± 8.7	23.5 ± 8.8	22.9 ± 8.4	0.4903	22.6 ± 8.5	23.2 ± 8.5	0.9669

*BMI* body mass index, *DL* dyslipidemia, *DM* diabetes mellitus, *euroSCORE II* European system for cardiac operative risk evaluation, *HD* renal failure requiring dialysis, *HT* hypertension, *IABP* intra-aortic balloon pumping, *JapanSCORE* Japanese system for cardiac operative risk evaluation, *LMT* left main trunk, *TVD* triple vessel disease, *LVEF* left ventricular ejection fraction, *OMI* old myocardial infarction, *ONCAB* on-pump coronary artery bypass grafting, *OPCAB* off-pump coronary artery bypass grafting, *PAD* peripheral arterial disease, *PCI* percutaneous coronary intervention, *STS score* American system for cardiac operative risk evaluation

### Operative findings and postoperative graft patency

The operative findings and postoperative graft patency are shown in Table 3. Complete revascularization occurred in all cases. Distal anastomoses were performed individually or by employing a sequential technique. In terms of sequential anastomosis, the LITA, RITA, GEA, and RA were used in 113 of 395 (29%), 10 of 327 (3%), 54 of 215 (25%), and 11 of 47 (23%) cases, respectively. One case involved using the RITA extended with the RA as a composite graft due to heavy calcification of the ascending aorta. After PSM, significant differences were observed in the utilization rates of sequential grafts using the GEA (OPCAB: 35% vs. ONCAB: 6%, *p* = 0.0117) and RA (OPCAB: 78% vs. ONCAB: 29%, *p* = 0.0211). However, the number of distal anastomoses (OPCAB: 3.0 ± 1.0 vs. ONCAB: 2.9 ± 0.9, *p* = 0.3865) and conduits used were not significantly different between the two groups.

**Table 3:** Operative findings and postoperative graft patency

		Matched cohort		
	All (*n* = 401)	OPCAB (*n* = 88)	ONCAB (*n* = 88)	*p*-value
No. of distal anastomosis	3.0 ± 1.0	3.0 ± 1.0	2.9 ± 0.9	0.3865
Complete revascularization	401 (100)	88 (100)	88 (100)	1.0000
Conduits used				
LITA	395 (99)	88 (100)	87 (98)	0.3159
RITA	327 (82)	71 (81)	69 (78)	0.7086
GEA	215 (54)	48 (55)	46 (52)	0.7625
RA	47 (12)	9 (10)	14 (16)	0.2635
Sequential graft				
LITA	113 (29)	27 (31)	24 (28)	0.6523
RITA	10 (3)	7 (10)	2 (3)	0.0932
GEA	54 (25)	17 (35)	6 (13)	0.0117
RA	11 (23)	7 (78)	4 (29)	0.0211
Postoperative graft patency				
CAG	328 (82)	73 (83)	73 (83)	1.0000
Graft patency for all patients	303 (92)	81 (92)	81 (92)	1.0000
Graft patency for all anastomoses	1161 (98)	257 (97)	244 (97)	0.9215
Complications (within 30 days)				
Mortality	2 (1)	0 (0)	2 (2)	0.1549
MACCE	38 (9)	7 (8)	13 (15)	0.1541
Cerebral infarction	4 (1)	1 (1)	1 (1)	0.7732
Mediastinitis	1 (1)	0 (0)	0 (0)	–
Re-exploration	6 (1)	0 (0)	3 (3)	0.0806

*CAG* coronary angiography, *GEA* gastroepiploic artery, *LITA* left internal thoracic artery, *MACCE* major adverse cardiac and cerebrovascular events, *ONCAB* on-pump coronary artery bypass grafting, *OPCAB* off-pump coronary artery bypass grafting, *RA* radial artery, *RITA* right internal thoracic artery

Postoperative coronary angiography was performed after a mean postoperative duration of 13.5 ± 8.7 days. Angiography was performed in 83% of patients who underwent OPCAB (*n* = 73) and ONCAB (*n* = 73). Graft patency for all patients (OPCAB: 92% vs. ONCAB: 92%, *p* = 1.0000) and all anastomoses (OPCAB: 97% vs. ONCAB: 97%, *p* = 0.9215) and 30-day mortality (OPCAB: 0% vs. ONCAB: 2%, *p* = 0.1549) were not significantly different between the two groups.

The details of postoperative graft patency are shown in Table 4. After PSM, graft patency for each graft and target was not different between the two groups.

**Table 4:** Details of postoperative graft patency

	Matched cohort						
	OPCAB (*n* = 88)			ONCAB (*n* = 88)			*p*-value
	Anastomosis	CAG	Graft patency	Anastomosis	CAG	Graft patency	
Graft							
LITA	87	73	71 (97)	87	72	68 (94)	0.3947
RITA	74	59	59 (100)	69	55	54 (98)	0.2982
GEA	50	43	38 (88)	46	38	36 (95)	0.3090
RA	9	9	9 (100)	14	12	12 (100)	–
Target							
LAD	88	73	72 (99)	85	70	70 (100)	0.3258
Dx	29	26	26 (100)	24	18	18 (100)	–
LCX	86	75	74 (99)	80	65	60 (92)	0.0639
RCA	62	55	50 (91)	63	53	51 (96)	0.2618

*CAG* coronary angiography, *Dx* diagonal branch, *GEA* gastroepiploic artery, *LAD* left anterior descending coronary artery, *LCX* left circumflex coronary artery, *LITA* left internal thoracic artery, *ONCAB* on-pump coronary artery bypass grafting, *OPCAB* off-pump coronary artery bypass grafting, *RA* radial artery, *RCA* right coronary artery, *RITA* right internal thoracic artery

The 30-day complications, including mortality, MACCEs, cerebral infarction, mediastinitis, and re-exploration, significantly differed between the two groups after PSM.

### Primary endpoint

In total, 79.6% (*n* = 319) of patients were followed up for a mean duration of 7.9 ± 6.3 years. Figure 1 shows a matched comparison of the cumulative survival rates between the OPCAB and ONCAB groups, demonstrating no significant difference (OPCAB hazard ratio, 1.04; 95% confidence interval, 0.53–2.04; *p* = 0.9138).

### Secondary endpoint

**Fig. 2:**
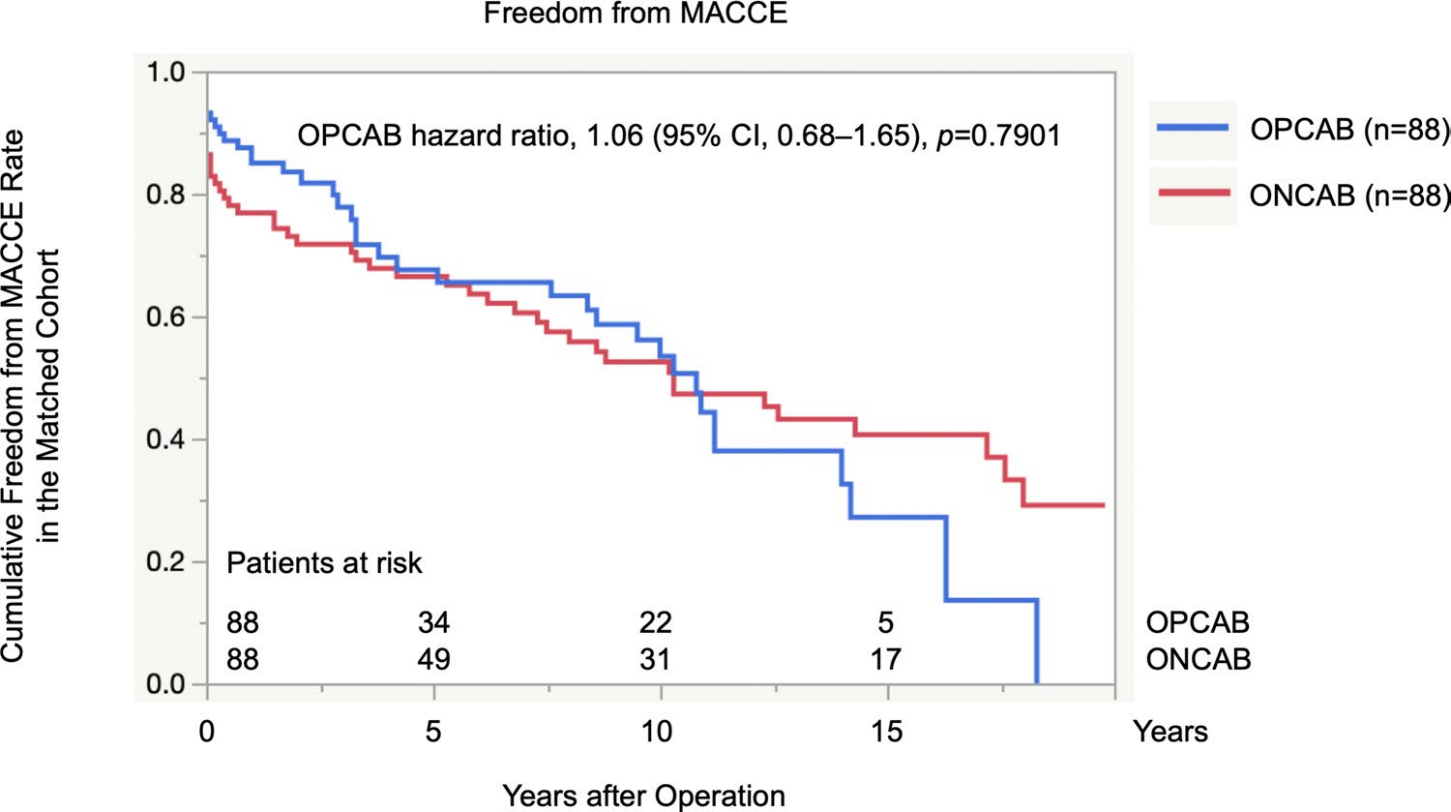
Cumulative rates of freedom from MACCEs in the matched cohort. The panel shows the cumulative rates of freedom from major adverse cardiac and cerebrovascular events in the matched cohort. The hazard ratios of the off-pump group compared to those of the on-pump group are shown. *MACCEs* major adverse cardiac and cerebrovascular events, *OPCAB* off-pump coronary artery bypass grafting, *ONCAB* on-pump coronary artery bypass grafting

Figure 2 shows a matched comparison of the cumulative rates of freedom from MACCEs between the OPCAB and the ONCAB groups, demonstrating no significant difference (OPCAB hazard ratio, 1.06; 95% confidence interval, 0.68–1.65; *p* = 0.7901).

### Risk analysis

Table 5 shows the results of the univariate and multivariate analyses for all-cause mortality. Based on the univariate analysis, previous stroke, renal failure requiring dialysis, LVEF ≤ 35%, and IABP were significantly associated with mortality. Multivariate analysis for mortality revealed that previous stroke and IABP were not independently associated with mortality. In contrast, renal failure requiring dialysis (hazard ratio, 4.67; 95% confidence interval, 2.48–8.79; *p* < 0.0001) and LVEF ≤ 35% (hazard ratio, 2.00; 95% confidence interval, 1.11–3.62; *p* = 0.0219) were independently associated with mortality.

**Table 5:** Risk factors for mortality

Univariate analysis (Cox proportional hazards)				
Factor	Survival (*n* = 333)	Death (*n* = 68)	*p*-value	Hazard ratio (95% CI)
Age ≥ 70 years	100 (30)	27 (40)	0.0973	1.51 (0.93–2.46)
Male	278 (83)	62 (91)	0.1115	1.98 (0.85–4.57)
BMI ≥ 30 kg/m^2^	20 (6)	1 (1)	0.3482	0.39 (0.05–2.80)
OMI	130 (39)	44 (65)	0.0581	1.62 (0.98–2.67)
PCI	63 (19)	12 (18)	0.9421	0.98 (0.52–1.83)
Stroke	27 (8)	13 (19)	0.0153	2.12 (1.15–3.88)
PAD	23 (7)	2 (3)	0.5689	0.66 (0.16–2.72)
HD	37 (11)	16 (24)	< 0.0001	5.22 (2.88–9.48)
HT	149 (45)	33 (49)	0.4815	1.18 (0.74–1.91)
DM	171 (51)	37 (54)	0.7001	1.10 (0.68–1.77)
Insulin	20 (6)	7 (10)	0.1784	1.71 (0.78–3.76)
DL	138 (41)	28 (41)	0.0668	0.63 (0.39–1.03)
LVEF ≤ 35%	37 (11)	19 (28)	< 0.0001	3.09 (1.81–5.29)
IABP	62 (19)	26 (38)	0.0240	1.77 (1.08–2.90)
LMT	70 (21)	11 (16)	0.6319	0.85 (0.45–1.63)
TVD	175 (53)	43 (63)	0.1733	1.41 (0.86–2.31)

Multivariate analysis (Cox proportional hazards)			
Factor	Hazard ratio	95% CI	*p*-value
Stroke	1.67	0.90–3.08	0.1030
HD	4.67	2.48–8.79	< 0.0001
LVEF ≤ 35%	2.00	1.11–3.62	0.0219
IABP	1.59	0.93–2.72	0.0918

*BMI* body mass index, *DL* dyslipidemia, *DM* diabetes mellitus, *HD* renal failure requiring dialysis, *HT* hypertension, *IABP* intra-aortic balloon pumping, *LMT* left main trunk, *LMT* triple vessel disease, *LVEF* left ventricular ejection fraction, *OMI* old myocardial infarction, *PAD* peripheral arterial disease, *PCI* percutaneous coronary intervention

Table 6 shows the results of univariate and multivariate analyses for MACCE. Based on the univariate analysis results, renal failure requiring dialysis and LVEF ≤ 35% were significantly associated with mortality. The results of the multivariate analysis for MACCEs revealed that LVEF ≤ 35% was not independently associated with MACCEs. Contrastingly, renal failure requiring dialysis was independently associated with MACCEs (hazard ratio, 2.23; 95% confidence interval, 1.42–3.50; *p* = 0.0005).

**Table 6:** Risk factors for major adverse cardiac and cerebrovascular events

Univariate analysis (Cox proportional hazards)				
Factor	MACCE (+) (*n* = 165) (%)	MACCE (−) (*n* = 236) (%)	*p*-value	Hazard ratio (95% CI)
Age ≥ 70 years	47 (28)	80 (34)	0.4629	0.88 (0.63–1.24)
Male	142 (86)	198 (84)	0.7602	1.07 (0.69–1.67)
BMI ≥ 30 kg/m^2^	7 (4)	14 (6)	0.5924	0.81 (0.38–1.74)
OMI	90 (55)	84 (36)	0.4834	1.12 (0.82–1.52)
PCI	34 (21)	41 (17)	0.5078	1.14 (0.78–1.66)
Stroke	21 (13)	19 (8)	0.2079	1.34 (0.85–2.13)
PAD	11 (7)	14 (6)	0.2206	1.47 (0.79–2.72)
HD	25 (15)	28 (12)	0.0003	2.23 (1.44–3.46)
HT	90 (55)	92 (39)	0.1468	1.26 (0.92–1.71)
DM	91 (55)	117 (50)	0.5167	1.11 (0.81–1.50)
Insulin	13 (8)	14 (6)	0.2551	1.39 (0.79–2.46)
DL	76 (46)	90 (38)	0.1670	1.25 (0.91–1.70)
LVEF ≤ 35%	29 (18)	27 (11)	0.0319	1.56 (1.04–2.33)
IABP	51 (31)	37 (16)	0.0620	1.37 (0.98–1.91)
LMT	32 (19)	49 (21)	0.9666	0.99 (0.67–1.46)
TVD	87 (53)	131 (56)	0.5938	0.92 (0.68–1.25)

Multivariate analysis (Cox proportional hazards)			
Factor	Hazard ratio	95% CI	*p*-value
Stroke	1.18	0.74–1.88	0.4915
HD	2.23	1.42–3.50	0.0005
LVEF ≤ 35%	1.26	0.82–1.93	0.3010
IABP	1.35	0.95–1.93	0.0962

*BMI* body mass index, *OMI* old myocardial infarction, *DL* dyslipidemia, *DM* diabetes mellitus, *HD* renal failure requiring dialysis, *HT* hypertension, *IABP* intra-aortic balloon pumping, *LMT* left main trunk, *LMT* triple vessel disease, *LVEF* left ventricular ejection fraction, *MACCE* major adverse cardiac and cerebrovascular events, *PAD* peripheral arterial disease, *PCI* percutaneous coronary intervention

## Discussion

The SYNTAX trial revealed that CABG was more effective in repairing complex coronary artery regions than PCI [1–3] and that survival outcomes were better with arterial grafts than with SVGs [4]. However, most previous studies reported only mid-term results for mortality [4–8]. The present study showed the long-term survival rate after total arterial CABG with either the OPCAB or ONCAB technique with complete revascularization. Additionally, the study examined details of postoperative graft patency, 30-day complications, and risk factors for mortality and MACCEs.

The currently published evidence on long-term outcomes after OPCAB compared to ONCAB remains controversial [9–15]. No significant difference in all-cause mortality and MACCE incidence was observed between the two groups in this study. The 5-year survival rates reported in this study (OPCAB: 87.7% vs. ONCAB: 91.6%, *p* = 0.9164) were favorable relative to the outcomes in the ROOBY (OPCAB: 84.8% vs. ONCAB: 88.1%, *p* = 0.02) [9, 10], CORONARY (OPCAB: 85.4% vs. ONCAB: 86.5%, *p* = 0.30) [11, 12], GOPCABE (OPCAB: 69% vs. ONCAB: 70%, *p* = 0.71) [13], and SYNTAX [1, 2] (CABG: 89.9% vs. PCI: 91.1%, *p* = 0.64) trials. Furthermore, the 10-year survival rates reported in this study (OPCAB: 87.7% vs. ONCAB: 79.4%, *p* = 0.9164) were more favorable than those in the SYNTAX [3] (CABG: 76% vs. PCI: 72%,* p* = 0.066) trial. This study showed that total arterial OPCAB and ONCAB had similar survival rates. The favorable long-term mortality outcomes observed in this study were probably due to the total arterial CABG with better patency grafts [6] and complete revascularization.

Several studies have reported that arterial grafts have better patency compared to SVGs. Some of the existing literature addressing the superior outcomes of multiple arterial versus traditional CABG has reported the decreased progression of native vessel disease in coronary territories revascularized with arterial grafts compared with SVGs as an explanatory factor [16]. This finding was based on the review of a large amount of coronary recatheterization data in patients treated with CABG, and the relatively greater release of nitric oxide from arterial versus SVG tissues has been suggested as the mechanism for this protection of native coronary beds against atherosclerosis progression [16, 17].

Indeed, the present study only observed short-term patency. However, especially regarding arterial grafts, given that the short-term patency is confirmed, it is reasonable to assume that long-term patency could be expected and also ensured [18–20]. Before PSM, significant differences in OMI, HT, and LVEF ≤ 35% were observed between the two groups. These factors were predictors of adverse long-term outcomes after CABG [21–25]. However, these differences were mitigated after propensity score matching, and the study specifically focused on comparing OPCAB and ONCAB.

The important similarity between the two groups in this study was the achievement of complete revascularization. Incomplete revascularization has a detrimental impact on long-term mortality [26, 27]. The ROOBY trial [9, 10] reported that a smaller number of anastomoses (OPCAB: 2.9 ± 0.9 vs. ONCAB: 3.0 ± 1.0, *p* = 0.002) and a higher rate of graft failure (OPCAB: 17.4% vs. ONCAB: 12.2%, *p* < 0.001) were associated with poorer outcomes in patients who underwent OPCAB. The present study shows that complete revascularization is paramount for better long-term outcomes, either with OPCAB or ONCAB.

OPCAB is technically demanding in terms of complete revascularization and patency [9, 10]. This study showed that OPCAB and ONCAB had a similar number of distal anastomoses and patencies. The postoperative graft patency details were observed to compare each graft and target. After PSM, graft patency for each graft and target was not different between the two groups.

Additionally, the existing literature showed that OPCAB had less postoperative morbidity at 30 days [11, 28]. However, this difference was not statistically significant after PSM.

This study demonstrated that renal failure requiring dialysis was a common finding after CABG, with increased mortality and MACCEs, similar to the observations in the CREDO-Kyoto cohort [29]. Furthermore, the present study observed that LVEF ≤ 35% was an independent risk factor for mortality, as reported in the STICH trial [30]. In summary, complete revascularization achieved through arterial grafts, with long-term patency assured, is expected to improve long-term outcomes, regardless of whether OPCAB or ONCAB is employed.

This clinical study had certain limitations. First, it was a retrospective observational study. Second, the results are susceptible to selection bias, as demonstrated by the differences in baseline characteristics, even after a propensity score-matched comparison. Third and most importantly, the follow-up rate was relatively lower than intended. Two reasons for this phenomenon exist: (i) Many patients from all over Japan who visited our facility were enrolled; hence, long-term follow-up of distant patients was difficult, and (ii) most Japanese families are nuclear; hence, many patients relocated during the follow-up period. Based on these limitations, comparing these results to other studies may be insufficient. Hence, additional studies with a longer follow-up and larger data sets may be required for further analysis. Nonetheless, this is currently one of the longest follow-up studies with PSM on total arterial CABG from a single institution in Japan.

In conclusion, total arterial OPCAB and ONCAB with complete revascularization showed similar rates of graft patency, survival, and MACCE incidence using PSM. Furthermore, renal failure requiring dialysis was observed to be a significant risk factor for mortality and MACCEs.

## Data Availability

Derived data supporting the findings of this study are available from the corresponding author upon reasonable request.
